# From Biomarker Discovery to Clinical Applications of Metabolomics in Glioblastoma

**DOI:** 10.3390/metabo15050295

**Published:** 2025-04-29

**Authors:** Neja Šamec, Gloria Krapež, Cene Skubic, Ivana Jovčevska, Alja Videtič Paska

**Affiliations:** Centre for Functional Genomics and Biochips, Institute of Biochemistry and Molecular Genetics, Faculty of Medicine, University of Ljubljana, Zaloška Cesta 4, 1000 Ljubljana, Slovenia; neja.samec@mf.uni-lj.si (N.Š.); gloria.krapez@mf.uni-lj.si (G.K.); cene.skubic@mf.uni-lj.si (C.S.); ivana.jovcevska@mf.uni-lj.si (I.J.)

**Keywords:** omics, metabolome, multiomic integration, tumor metabolic reprogramming, biomarker, diagnosis, therapy, precision oncology

## Abstract

Background/Objectives: In recent years, interest in studying changes in cancer metabolites has resulted in significant advances in the metabolomics field. Glioblastoma remains the most aggressive and lethal brain malignancy, which presents with notable metabolic reprogramming. Methods: We performed literature research from the PubMed database and considered research articles focused on the key metabolic pathways altered in glioblastoma (e.g., glycolysis, lipid metabolism, TCA cycle), the role of oncometabolites and metabolic plasticity, and the differential expression of metabolites in glioblastoma. Currently used metabolomics approaches can be either targeted, focusing on specific metabolites and pathways, or untargeted, which involves data-driven exploration of the metabolome and also results in the identification of new metabolites. Data processing and analysis is of great importance and can be improved with the integration of machine learning approaches for metabolite identification. Results: Changes in α/β-glucose, lactate, choline, and 2-hydroxyglutarate were detected in glioblastoma compared with non-tumor tissues. Different metabolites such as fumarate, tyrosine, and leucine, as well as citric acid, isocitric acid, shikimate, and GABA were detected in blood and CSF, respectively. Conclusions: Although promising new technological and bioinformatic approaches help us understand glioblastoma better, challenges associated with biomarker availability, tumor heterogeneity, interpatient variability, standardization, and reproducibility still remain. Metabolomics research, either alone or combined with genomics or proteomics (i.e., multiomics) in glioblastoma, can lead to biomarker identification, tracking of metabolic therapy response, discovery of novel metabolites and pathways, and identification of potential therapeutic targets.

## 1. Introduction

### 1.1. Metabolomics

Metabolomics is the quantitative profiling of the endogenous metabolites found in biofluids to characterize the metabolic phenotype of a disease or assess its response to stimuli [1,2]. Metabolic alterations in body fluids and tissues can improve clinical diagnosis and the restoration of cellular equilibrium after drug exposure, but they can also highlight off-target effects and potential toxicity. Therefore, identification of new metabolic biomarkers can open new paths in the discovery and development of new diagnostic and/or therapeutic strategies [2]. Metabolites are small molecules, <1500 Da in size, that represent the metabolic pathways in the tissues [3]. The relationship between the metabolism and cancer was proposed in the 1920s by Otto von Warburg, who showed that cancer cells have increased glycolysis rates and decreased dependence on oxidative phosphorylation even in the presence of oxygen [4,5]. His theory evolved into what is today known as the “Warburg effect”, which refers to the alteration in the use and synthesis of important metabolites, e.g., glucose and fatty and amino acids, by tumor cells [5,6]. Even in aerobic conditions, tumor cells prefer to transform glucose to lactate as the main energy source, as it is the most time-effective. This aerobic glycolysis is one of the most understood metabolic adaptations of tumor cells. It is clear today that metabolic reprogramming is one of the hallmarks of cancer [7]. Therefore, the identification of metabolites that can be used for diagnosis or as targets for therapy has long been at the center of cancer research. Metabolic profiling of biofluids (urine and sera) is used to visualize the metabolites of patients with cancer and gastroenterological disease [8], while metabolic profiling of tissue specimens has revealed variations between metabolites in tumor and reference samples [6,9]. The brain is one of the most metabolically active organs in the human body [10]. Glucose is the main energy source for the brain, consuming about 20% of the daily body use [7].

### 1.2. The Metabolic Profile of Glioblastoma

Even though primary brain tumors are considered rare and represent 1–2% of human cancers, 8–10 cases per 100,000 inhabitants are diagnosed in Europe every year [11]. The majority (80.8%) of primary brain tumors are gliomas classified into grades I to IV by the WHO. Of these, 57.7% are grade IV glioblastomas, which have an incidence of 3.23 per 100,000 population [7]. Glioblastoma is still one of the most lethal cancers, with poor patient prognosis and survival of 8 to 14 months after diagnosis. Treatment is multimodal and aggressive, consisting of surgical removal of the tumor mass, chemotherapy with temozolomide, and radiation. Even with the most recent scientific and technological advances, the 5-year survival is only 5.6% [3]. Poor patient survival and lack of more efficient therapeutic strategies are a result of the great cellular and molecular heterogeneity of glioblastoma, which is detected at genetic, transcriptional, metabolic, and functional levels [7]. In addition to the four genetic glioblastoma subtypes as described by Verhaak et al. [12], Chinnaiyan et al. identified three metabolic subclasses of glioblastoma, named energetic, anabolic, and phospholipid catabolism, with prognostic relevance [13].

In order to support their bioenergetic and biosynthetic needs for tumor development, growth, and invasion, glioblastomas have altered metabolisms [14]. Multiple pathways including oxidative phosphorylation (OXPHOS), the pentose–phosphate pathway (PPP), fatty acid (FA) biosynthesis and oxidation, and nucleic acid biosynthesis are changed in order to support the fast tumor growth. The end result of these changes is the use of glucose as an energy source and the promotion of FA synthesis for membrane biogenesis [15]. However, in the absence of glucose, i.e., glucose deprivation, glial cells are able to use ketone bodies (KBs) and FAs as fuel [15]. In addition, increased glycolysis flow, lipid storage, and activation of PI3K/Akt/mTOR signaling are also detected in glioblastomas. Clinically, it is known that choline and lactic acid are elevated in malignant gliomas compared with normal brain tissue. What is more, grades III and IV tumors have higher lactic acid levels, and grade IV has significantly elevated lipids [6]. Differences in metabolite composition are also observed between the glioblastoma non-invasive core and the invasive margin. Wood et al. showed the intratumor metabolic heterogeneity of glioblastoma by analyzing 4–5 tumor regions per patient (5 patients total) and obtaining metabolomic and lipidomic profiles [16]. Similar observations were reported by He et al., who used OrbiSIMS and LESA-MS/MS for untargeted metabolomics to probe intratumoral heterogeneity in glioblastoma [17]. They showed that different subpopulations of cells that can be found within tumors, such as necrotic, viable and non-cancerous, have distinct metabolic profiles. Metabolic differences between glioblastoma and peritumoral tissues were identified in the study by Kampa et al. [3]. The authors performed mass spectrometry imaging (MSI) on 25 thin sections from resected tumors and found increased levels of antioxidants (ascorbic acid, taurine, and glutathione) and purine and pyrimidine metabolism compounds in tumor areas. They also reported decreased *N*-acetylaspartate (NAA), which is a marker for neuronal health. In addition, cerebrospinal fluid (CSF) from patients with malignant gliomas may present with a signature of altered metabolism [18]. Analyzing CSF from glioma patients, Nakamizo et al. detected 61 metabolites. Of these, lactic acid levels in the CSF of glioblastoma patients were significantly higher when compared with glioma grades I and II. In their study, higher lactic acid levels were detected in *IDH*-mutant than in *IDH*-wildtype grade I–III gliomas. Higher lactic acid levels are associated with shorter overall survival (OS) in malignant gliomas. Tricarboxylic acid (TCA) cycle metabolites, citric and isocitric acid, were also elevated in glioblastomas compared with grade I–III gliomas [6].

While oncogenes and tumor suppressors (e.g., p53, PI3KCA) contribute to cell regulation, little is known about the mutations in genes encoding metabolic enzymes (e.g., succinate dehydrogenase (*SDH*), fumarate dehydrogenase, and isocitrate dehydrogenase (*IDH*)) and their contribution to oncogenesis [19].

Magnetic resonance imaging (MRI) is still the gold standard for glioblastoma diagnosis, but it faces challenges in distinguishing between tumor-like brain lesions and glioblastoma or recurrent tumors and radionecrosis. MRI can be improved with in vivo magnetic resonance spectroscopy (MRS). MRS can provide metabolic information about the tumors such as through detection of NAA, choline-containing compounds (ChoCCs), and creatine (Cr), and in some cases, also glutamate, glutamine, lactate, and alanine [11]. Moreover, ex vivo high-resolution magic-angle-spinning nuclear magnetic resonance (HR-MAS NMR) metabolomics studies can help in discriminating glioma grades and in the development of new clinical strategies. Because of the higher resolution of ex vivo HR-MAS NMR, ten times as many metabolites are found compared with those recognized in vivo [11]. Despite the advances in imaging techniques, and even with the use of AI (artificial intelligence i.e., machine and deep learning) for image analysis, there is still no FDA-approved biomarker for glioblastoma diagnosis [20]. So, the need for non-invasive, reliable, and accessible biomarkers for glioblastoma diagnosis and therapy remains unmet.

In this regard, Ferassi et al. evaluated the metabolic profile of blood plasma from glioblastoma patients and compared it with that of healthy controls [21]. The authors detected increased levels of seven metabolites in the plasma of glioblastoma patients, in particular pyruvate, 5-hydroxymethyluracil, arginyl-proline, phosphatidylserine, 3-O-sulfogalactosylceramide, 3-oxodecanoyl-CoA, and N-acylphosphatidylethanolamine (NAPE) and proposed them as chemical markers for glioblastoma. Similarly, Bobeff et al. analyzed amino acid levels in the plasma of 18 glioblastoma patients and compared them with 15 controls [22]. The authors detected lower amino acid levels in the glioblastoma samples compared with control, especially for plasma levels of arginine, glutamic acid, glutamine, glycine, and histidine. In their study, the only amino acid with a higher median plasma level in glioblastoma samples was aspartic acid.

The objective of this review is to provide deeper insight into the opportunities that the rapidly evolving field of metabolomics can offer to glioblastoma patients in terms of better diagnosis and treatment. As the knowledge of glioblastoma metabolic reprogramming and molecular mechanisms increases, modern and innovative therapeutic patient-tailored strategies can be developed.

## 2. Metabolic Reprogramming in Glioblastoma

**Figure 1:**
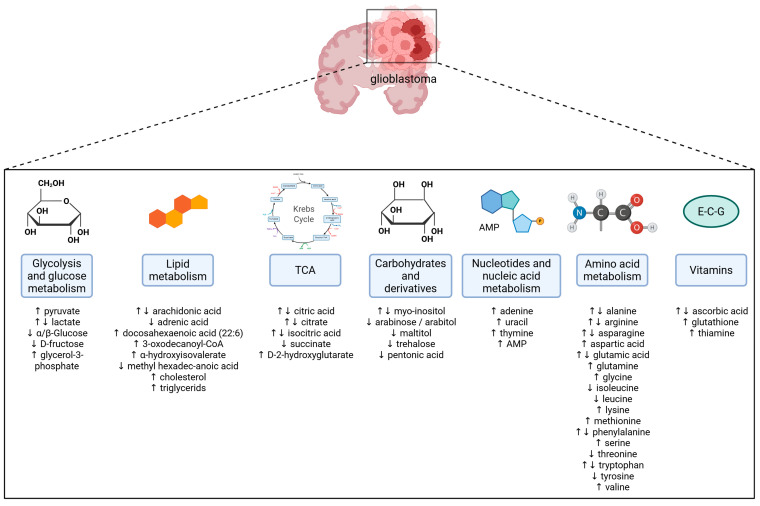
Alterations in specific metabolites in glioblastoma. ↑ denotes increased levels and ↓ denotes decreased levels of a specific metabolite. For more detailed information, please refer to Table 1, Table 2, Table 3 and Table 4. Created in BioRender. Videtič Paska, A. (2025) https://BioRender.com/lt41n2m.

This section is an overview of the current literature summarizing metabolites discovered in human samples, namely plasma, tumor tissue, and CSF. Articles were obtained using the PubMed platform. Primary search terms included “glioblastoma” and “metabolomics” or “metabolites”. At the time of our research, 116 articles corresponding to these search terms were available. Most of these articles were published in the past 5 years. We went through all of them, excluding reviews and in vitro research papers. We included only research papers utilizing metabolomics in screening of human samples, with the result that we identified statistically significant metabolites found in 23 relevant research papers. Most of the articles sampled tumor tissues from glioblastoma patients, but six of them sampled plasma [21,23,24,25,26,27], and three considered CSF [6,28,29]. To further strengthen our research, additional papers, specific for individual metabolites utilizing targeted metabolomics, were used and their findings included. With this research, we aimed to identify standout metabolites that could be used as novel glioblastoma-specific biomarkers. An overview of the changed metabolites is presented in Figure 1.

We especially wanted to identify metabolic biomarkers from the CSF and plasma. We believe that the current data on metabolites need to be expanded to find a robust set of glioblastoma biomarkers. Based on the scientific paper growth chart of this subject, we believe that the field is still evolving, and more comprehensive studies researching glioblastoma metabolomes will be published in the near future, and with that, robust metabolite biomarkers for glioblastoma will be identified.

### 2.1. Glycolysis, Ketone Bodies, and TCA

The adult brain meets its energy requirements by the oxidation of glucose-producing pyruvate and converting it into acetyl-CoA, which then feeds the TCA cycle and supports the electron transfer chain. This results in efficient ATP production and little lactic acid production [15]. Glioblastoma, however, is associated with a shift toward glycolysis, even under aerobic conditions [6,21,23,28,30], which drastically increases its lactate production. Glioblastoma can increase its biomass production and invasion by acidification of the tumor microenvironment (TME). Increases in lactate and pyruvate production were shown to be robust biomarkers for glioblastoma (Table 1) [3,6,23,24,28]. Elevated lactate levels were observed in CSF, plasma, and tumor tissues, correlating with poor overall survival (OS) and specific genetic profiles such as TP53-wildtype glioblastoma [3,6,23,24,28]. Because of the high energy requirements of glioblastoma, most energy-associated metabolites like α/β-glucose are quickly depleted, which is another characteristic of this disease. The most important role of glucose metabolism is supporting glioblastoma proliferation, survival, and its invasive phenotype [23,31]. Different methods are utilized for monitoring such metabolites. The most common ones are gas chromatography–mass spectrometry (GC-MS), liquid chromatography–tandem mass spectrometry (LC-MS/MS), mass spectrometry imaging (MSI), and nuclear magnetic resonance (NMR) spectroscopy. These consistently detect alterations in glycolysis and glucose metabolism across different biological samples [3,6,21,23,24,28,31,32]. These metabolites also highlight potential metabolic biomarkers for diagnosis and therapeutic intervention.

**Table 1:** Glycolysis, ketone bodies, and TCA metabolic pathway alterations and metabolic biomarker profiles in glioblastoma. Overview of recent metabolomics studies performed on tumor tissue and plasma and CSF samples.

Metabolic Pathway	Metabolites	Sample Type	Identification Method	GBM Specificity	Ref.
Glycolysis and glucose metabolism	Pyruvate	Plasma CSF	ESI-LTQ-MS H-NMR GC-MS	↑ IDH-wt GBM vs. IDH-mut astrocytoma ↑ glioma patients vs. healthy individuals	[6,21,23,24]
	Lactate	CSF Tumor Plasma	MALDI-TOF-MSI H-NMR GC-MS LC-MS/MS-SRM	↑ TP53-wt vs. TP-mut GBM ↑ GBM vs. peritumoral tissue ↓ Glioma patients vs. healthy individuals ↑ Glioma patients vs. healthy individuals ↓ IDH-wt vs. IDH-mut GBM ↑ associated with poor OS in grade IV gliomas	[3,6,23,24,28]
	α/β-glucose	Plasma	H-NMR	↓ Glioma patients vs. healthy individuals	[23]
	D-Fructose	Tumor	GC-MS	↓ GBM vs. peritumoral tissue	[31]
	Glycerol-3-phosphate	Tumor	IMAC–SRM LC/GC-MS	↑ IDH-mut grade IV astrocytoma vs. IDH-wt GBM ↑ invasive vs. non-invasive regions of GBM	[32,33]
Tricarboxylic acid cycle	Citric acid	CSF	GC-MS	↑ GBM vs. low-grade glioma ↓ IDH-wt vs. IDH-mut grade IV astrocytoma	[6]
	Citrate	Tumor Plasma	MALDI-TOF-MSI H-NMR	↑ GBM vs. peritumoral tissue ↓ glioma patient vs. healthy individual ↓ GBM vs. low-grade gliomas	[3,13,23]
	Isocitric acid	CSF	GC-MS	↑ GBM vs. low-grade gliomas ↓ IDH-wt vs. IDH-mut grade IV astrocytoma	[6]
	Succinate	Tumor	LC-MS/MS	↓ IDH-wt GBM vs. healthy individuals	[13,34]
	D-2-hydroxyglutarate	Tumor CSF	IMAC-SRM LC-MS/MS Enzymatic assay MALDI-TOF-MSI HR-NMR	↑ IDH-mut vs. IDH-wt grade IV astrocytoma	[3,13,29,32,34,35,36,37]
	Homocitrate	Tumor	LC-MS/MS	↓ IDH-wt GBM	[34]
	Itaconate	Tumor	LC-MS/MS	↓ IDH-wt GBM	[34]
	Malate	Tumor Plasma	LC-MS/MS GC-MS	↑ IDH-wt GBM ↑ Glioma patients vs. healthy individuals	[24,34]
	Fumarate	Plasma	GC-MS	↑ Glioma patients vs. healthy individuals	[24]
	α-ketoglutaric acid	Tumor	GC-MS	↑ GBM vs. healthy surrounding brain tissue	[31]
Ketone bodies	3-hydroxybutanoic acid	Tumor	GC-MS	↑ GBM vs. healthy surrounding brain tissue	[31]
	Ketovalerate	Tumor	LC-MS/MS	↑ IDH-wt GBM	[34]
Carbohydrates and derivatives	Myo-inositol	Tumor Plasma	IMAC-SRM H-NMR HR-MRS GC-MS	↑ IDH-mut grade IV astrocytoma vs. GBM ↓ Glioma patients vs. healthy individuals ↓ GBM patients vs. healthy individuals ↓ GBM vs. healthy surrounding brain tissue	[23,31,32,36,38,39]
	Arabinose/arabitol Maltitol Trehalose Pentonic acid	Tumor	GC-MS	↓ GBM vs. healthy surrounding brain tissue	[31]

↑ denotes increased levels and ↓ denotes decreased levels of a specific metabolite. GBM, glioblastoma; LC-MS/MS, liquid chromatography–tandem mass spectrometry; MS, mass spectrometry; SRM, selected reaction monitoring; ESI, electrospray ionization; LTQ, linear trap quadrupole; H-NMR, proton nuclear magnetic resonance; MALDI, matrix-assisted laser desorption/ionization; TOF, time of flight; MSI, mass spectrometry imaging; GC-MS, gas chromatography–mass spectrometry; IMAC, immobilized metal affinity chromatography; MRS, magnetic resonance spectroscopy; CSF, cerebrospinal fluid; IDH, isocitrate dehydrogenase; IDH-wt, isocitrate dehydrogenase wild-type; IDH-mut, isocitrate dehydrogenase mutant.

When comparing glioblastoma cells with normal brain cells, which can alternatively metabolize KBs as an energy source during glucose shortage, they often present with a reduced capacity to metabolize KBs [40]. This change in metabolism is a result of the downregulation of ketolytic enzymes such as 3-oxoacid-CoA transferase 1 and acetyl-CoA acetyltransferase 1. Because of this shift, glioblastoma cells are unable to utilize KBs like 3-hydroxybutanoic acid and ketovalerate for energy production [31,34]. Based on this metabolic specificity, ketogenic diets have been explored as a potential therapy in conjunction with other typical treatments, with varying degrees of success [40].

Another crucial cellular energy metabolism shift in glioblastoma happens in the TCA cycle. Mutations in IDH enzymes, particularly IDH1 and IDH2, lead to the production of the oncometabolite D-2-hydroxyglutarate (D-2HG). This aberrant metabolite inhibits α-ketoglutarate-dependent dioxygenases, resulting in epigenetic modifications that promote tumorigenesis [41]. Furthermore, glioblastoma cells can increase glutaminolysis, converting glutamine to glutamate and subsequently to α-ketoglutarate, replenishing TCA cycle intermediates and supporting anabolic growth. Glutamine and glutamate, as well as α-ketoglutarate, were mostly shown to be elevated in plasma and tumor samples [3,13,23,26,27,42]. With this metabolic reprogramming, glioblastoma cells acquire an increased cell proliferation [13]. High levels of TCA metabolites like fumarate and malate can also act as competitive inhibitors of prolyl hydroxylases. This, in turn, leads to the stabilization of hypoxia-inducible factors and promoting of a pseudohypoxic state that promotes tumor resistance to apoptosis and invasion. High levels of TCA metabolites also promote a stem-like phenotype and increase glycolysis production in glioblastoma [43]. Understanding the metabolic changes that happen in the TCA cycle could lead to new therapeutic approaches, like targeting metabolic enzymes specific to the TCA cycle or its associated pathways to disrupt the metabolic flexibility of glioblastoma cells.

### 2.2. Amino Acid Metabolism

Similar to glucose and energy metabolism, amino acid metabolism in glioblastoma also undergoes reprogramming that supports the tumor’s growth and survival. Numerous studies have shown that glioblastomas upregulate amino acid production as well as uptake amino acids from their surroundings. The individual amino acids are then used for protein synthesis, as precursors for nucleotide biosynthesis, and for synthesis of other macromolecules that help the glioblastoma to survive and proliferate [3,13,27]. Studies have reported elevated concentrations of amino acids such as serine, glycine, and proline modifications in glioblastoma, which are integral to one-carbon metabolism and nucleotide biosynthesis and more, as seen in Table 2 [26,27,31]. This metabolic shift promotes the anabolic pathways and proliferation characteristic of glioblastoma cells [44]. Among other amino acids, methionine seems to be of particular importance for the glioblastoma cells. Methionine is an amino acid involved in methylation reactions and polyamine synthesis [25,45]. By exploiting this metabolic specificity, we could inhibit methionine utilization, which is crucial for genome methylation and gene suppression. Widespread methylation depletion could enhance chromosomal instability and support the transformation of glioblastoma stem cells from a proneural to a mesenchymal state, leading to a more favorable glioblastoma prognosis [46]. Amino acid metabolic adaptations not only support the anabolic and energetic needs of glioblastoma cells but also contribute to the immunosuppressive tumor microenvironment by depleting amino acids that are crucial for immune cell function [44].

**Table 2:** Amino acid metabolism pathway alterations and metabolic biomarker profiles in glioblastoma. Overview of recent metabolomics studies performed on tumor tissue and plasma and CSF samples.

Molecules	Metabolites	Sample Type	Identification Method	GBM Specificity	Ref.
Amino acids	Valine	Plasma Tumor	LC-MS/MS H-NMR GC-MS	↑ GBM vs. healthy individuals ↑ Glioma patients vs. healthy individuals ↑ GBM vs. surrounding healthy brain tissue	[23,26,27,31]
	Alanine	Plasma Tumor	H-NMR GC-MS MRS	↓ Glioma patients vs. healthy individuals ↑ GBM vs. surrounding healthy brain tissue ↑ IDH-wt grade IV astrocytoma ↑ Associated with poor OS	[23,31,39]
	Tyrosine	Plasma	H-NMR	↓ Glioma patients vs. healthy individuals	[23]
	Leucine	Plasma	H-NMR LC-MS/MS	↓ Glioma patients vs. healthy individuals	[23,26]
	Isoleucine	Plasma	H-NMR LC-MS/MS	↓ Glioma patients vs. healthy individuals	[23,26]
	Asparagine	Plasma Tumor	LC-MS/MS	↓ GBM patients vs. healthy individuals ↑ IDH-wt GBM	[26,27,31,34]
	Serine	Plasma Tumor	LC-MS/MS GC-MS	↑ High levels associated with poor OS ↑ GBM vs. surrounding healthy brain tissue	[26,31]
	Taurine	Plasma Tumor	LC-MS/MS MALDI-FTICR-MS	↑ High levels associated with poor OS ↑ GBM patients vs. healthy individuals	[3,26]
	Citrulline	Plasma	LC-MS/MS	↑ associated with GBM progression and poor OS ↑ GBM patients vs. healthy individuals	[26,27]
	Glutamine	Plasma Tumor	LC-MS/MS MALDI-TOF-MS	↑ GBM patients vs. healthy individuals ↑ GBM vs. peritumoral tissue	[3,13,27]
	Lysine	Plasma Tumor	LC-MS/MS GC-MS	↑ GBM patients vs. healthy individuals ↑ GBM vs. healthy surrounding brain tissue	[27,31]
	Ornithine	Plasma Tumor	LC-MS/MS GC-MS	↑ GBM patients vs. healthy individual ↑ GBM vs. healthy surrounding brain tissue	[25,27,31]
	Threonine	Plasma	LC-MS/MS	↓ GBM patients vs. healthy individuals	[27]
	Tryptophan	Plasma Tumor	LC-MS/MS GC-MS	↓ GBM patients vs. healthy individuals ↑ GBM vs. healthy surrounding brain tissue	[27,31]
	Methionine	Plasma	LC-MS/MS	↑ IDH-mut vs. IDH-wt grade IV astrocytoma	[25,45]
	Arginine	Plasma	LC-MS/MS	↓ Associated with better OS ↑ Associated with high grade gliomas	[25,45]
	Phenylalanine	Plasma Tumor	H-NMR GC-MS	↓ Glioma patients vs. healthy individuals ↑ GBM vs. healthy surrounding brain tissue	[23,31]
	Aspartate	Tumor	HR-MAS	↑ Associated with better OS	[36]
	Glycine	Tumor	GC-MS	↑ GBM vs. healthy surrounding brain tissue	[31]
	Sarcosine	Plasma	LC-MS/MS	↑ Associated with IDH-mut GBM	[25]
Amino acid derivatives	N-Acetylaspartate	Tumor	MALDI-TOF-MSI HR-MAS	↓ GBM vs. peritumoral tissue ↓ GBM vs. low grade astrocytoma	[3,36]
	Glutamate	Plasma Tumor	LC-MS/MS H-NMR	↑ GBM patients vs. healthy individuals ↑ associated with poor OS ↓ Glioma patients vs. healthy individuals ↓ IDH-mut vs. IDH-wt grade IV astrocytoma	[23,26,42]
	Cystathionine	Tumor	LC-MS/MS GC-MS	↑ Invasive vs. non-invasive regions of GBM	[33]
	S-Methyl-L-cysteine	Tumor	GC-MS	↑ GBM vs. healthy surrounding brain tissue	[31]
	4-Hydroxyglutamate	Tumor	LC-MS/MS	↑ Associated with IDH-wt GBM	[34]
	Methylhistidine	Plasma	H-NMR	↓ Glioma patients vs. healthy individuals	[23]
	Allothreonine	Tumor	GC-MS	↑ GBM vs. healthy surrounding brain tissue	[31]
	Kynurenate	Plasma	LC-MS/MS	↑ High values associated with low OS	[45]
	3-Cyanoalanine	Tumor	GC-MS	↑ GBM vs. healthy surrounding brain tissue	[31]
	Arginyl-proline	Plasma	ESI-LTQ-MS	↑ Associated with IDH-wt GBM	[21]
	Pyroglutamic acid	Plasma	LC-MS/MS GC-MS	↓ Glioma patients vs. healthy individuals ↑ Associated with high grade gliomas	[24,25]
	Aminoadipate	Tumor	LC-MS/MS	↑ IDH-wt GBM	[34]
	4-Hydroxyphenylpyruvate	Tumor	LC-MS/MS	↑ IDH-wt GBM	[34]
	Cis-4-Hydroxyproline	Plasma	LC-MS/MS MALDI-TOF	↑ GBM patients vs. healthy individuals	[27]
	Trans-4-Hydroxyproline	Plasma	LC-MS/MS MALDI-TOF	↓ GBM patients vs. healthy individuals	[27]
Neurotransmitter-related amino acid derivatives	GABA	CSF	MRS	↑ TP53-wt vs. TP53-mut GBM ↑ PTEN-mut vs. PTEN-wt GBM	[42]
	5-Methoxytryptamine	Tumor	LC-MS/MS GC-MS	↑ Invasive vs. non-invasive regions of GBM	[33]
	Aminobutanal	CSF	LC-MS/MS-SRM	↑ Associated with poor OS	[42]
	Acetylcholine	CSF	LC-MS/MS-SRM	↑ Associated with poor OS	[28]
Polyamines	Putrescine	Plasma	LC-MS/MS MALDI-TOF	↑ GBM patients vs. healthy individuals	[27]
	Spermidine	Plasma Tumor	LC-MS/MS	↑ GBM patients vs. healthy individuals ↑ Invasive vs. core regions of GBM	[26,33]
	Spermine	Plasma	LC-MS/MS	↓ GBM patients vs. healthy individuals	[27]
	N-acetylputrescine	CSF Plasma	LC-MS/MS-SRM LC-MS	↑ GBM pre- vs. post-treatment ↑ Associated with IDH-wt GBM	[25,42]
Creatine related	Guanidoacetic acid	Plasma	LC-MS/MS	↑ Associated with IDH-wt GBM	[25]
	Creatinine	Tumor Plasma	LC-MS/MS H-NMR HR-MAS LC-MS	↓ Associated with IDH-wt GBM ↑ Associated with IDH-wt GBM ↑ Associated with invasive GBM borders ↓ Glioma patients vs. healthy individuals ↓ Associated with grade IV astrocytoma	[23,25,33,34,36]

↑ denotes increased levels and ↓ denotes decreased levels of a specific metabolite. GBM, glioblastoma; LC-MS/MS, liquid chromatography–tandem mass spectrometry; MS, mass spectrometry; SRM, selected reaction monitoring; ESI, electrospray ionization; LTQ, linear trap quadrupole; HR-MAS, high-resolution magic-angle-spinning; H-NMR, proton nuclear magnetic resonance; MALDI, matrix-assisted laser desorption/ionization; FTICR, Fourier transform ion cyclotron resonance; TOF, time of flight; MSI, mass spectrometry imaging; GC-MS, gas chromatography–mass spectrometry; MRS, magnetic resonance spectroscopy; CSF, cerebrospinal fluid; IDH, isocitrate dehydrogenase; IDH-wt, isocitrate dehydrogenase wild-type; IDH-mut, isocitrate dehydrogenase mutant; OS, overall survival.

### 2.3. Lipid Metabolism

Glioblastoma is also characterized by significant alterations in lipid metabolism that support tumor growth, survival, and resistance to therapies. These metabolic reprogramming events include increased FA synthesis, enhanced lipid uptake, and modifications in lipid composition, all of which contribute to the aggressive nature of glioblastoma [47]. One prominent alteration is the upregulation of FA synthase (FASN), the key enzyme responsible for de novo FA synthesis. Elevated FASN expression in glioblastoma leads to increased production of FAs, which are essential components for membrane biogenesis, energy storage, and signaling molecules that promote tumor proliferation and survival. Inhibition of FASN has been shown to sensitize glioblastoma cells to chemotherapy, indicating its potential as a therapeutic target [47,48]. FA oxidation is an important source of energy production. However, the brain does not use FAs for oxidative metabolism, but uses KBs instead [49]. During energy requirements, acetyl-CoA can be transferred to the mitochondria and peroxisomes, which together maintain lipid homeostasis. However, as the mitochondrial membrane is not permeable to acetyl-CoA, FAs must be conjugated to carnitine in order to enter the mitochondrion. Intracellularly, carnitine is accumulated by the OCTN2 carnitine transporter in the heart, muscles, and kidneys. Carnitine then forms bonds with long-chain FAs through carnitine palmitoyl transferase 1 (CPT-1). There are three CPT-1 isoforms, of which CPT-1A is expressed in the brain, among other organs. Bogusiewicz et al. investigated alterations in the carnitine shuttle system as an indication for cancer in particular gliomas [50]. The authors sampled brain tumors from 19 patients using solid-phase microextraction (SPME) immediately after excision. The analysis was performed on liquid chromatography–high-resolution mass spectrometry and resulted in the extraction of carnitine and 22 simple-chain saturated and unsaturated acylcarnitines. They reported slightly higher levels of carnitine in samples from high-grade gliomas compared with low-grade gliomas (a ratio of 4.21) and in the IDH wildtype compared with the IDH mutant (fold change of 3.91). Glioblastoma cells also exhibit increased expression of lipid transporters, such as CD36 and FA-binding proteins, facilitating the uptake of exogenous FAs from the TME. This supports the metabolic flexibility of glioblastoma cells, allowing them to adapt to varying nutrient availability and sustain rapid growth [51]. Alterations in lipid composition are evident in glioblastoma, with studies showing increased levels of total lipid content compared with normal brain tissues [26,27]. One such lipid is cholesterol, which was shown to be overexpressed in the tumor tissue [33] and plasma [27] of glioblastoma patients (Table 3).

**Table 3:** Lipid metabolism pathway alterations and metabolic biomarker profiles in glioblastoma. Overview of recent metabolomics studies performed on tumor tissue and plasma and CSF samples.

Molecules	Metabolites	Sample Type	Identification Method	GBM Specificity	Ref.
Fatty acids	Arachidonic acid	Tumor	MALDI-FTICR-MS GC/LC-MS/MS	↓ GBM vs. peritumoral tissue ↑ Higher in mesenchymal-like GBM subtype	[3,42]
	Adrenic acid	Tumor	MALDI-FTICR-MS	↓ GBM vs. peritumoral tissue	[3]
	Docosahexaenoic acid (22:6)	Tumor	GC/LC-MS/MS	↑ Higher in proneural-like GBM subtype	[42]
	3-oxodecanoyl-CoA	Plasma	ESI-LTQ-MS	↑ Associated with IDH-wt GBM vs. IDH-mut GBM	[21]
	α-hydroxyisovalerate	Plasma	GC-MS	↑ Glioma patients vs. healthy individuals	[24]
	Methyl hexadecanoic acid	Plasma	GC-MS	↓ Glioma patients vs. healthy individuals	[24]
Acylcarnitines	Carnitine	CSF Tumor Plasma	LC-MS/MS-SRM LC-MS/MS LC-MS/MS	↑ GBM pre- vs. post-treatment ↑ IDH-wt GBM ↑ P53-wt vs. P53-mut GBM	[25,27,28,34]
	Propionylcarnitine 2-methylbutyrylcarnitine Isobutyryl-L-carnitine Deoxycarnitine L-palmitoylcarnitine	CSF	LC-MS/MS-SRM	↑ GBM pre- vs. post-treatment ↑ P53-wt vs. P53-mut GBM	[28]
	Pymeloylcarnitine	Plasma	FIA-MS	↑ GBM patients vs. healthy individuals	[26]
	Hydroxyhexadecenoylcarnitine Hydroxyhexadecadienylcarnitine	Plasma	LC-MS/MS	↑ Associated with better OS	[26]
	Octanylcarnitine	Plasma	LC-MS/MS	↑ Associated with poor OS	[26]
	Stearoylcarnitine	Tumor	LC-MS/MS	↑ GBM patients vs. healthy individuals	[27]
Cholesterol and isoprenoids	Cholesterol	Tumor Plasma	LC-MS/MS	↑ Associated with EGFR activation in GBM ↑ GBM patients vs. healthy individuals ↑ Invasive vs. core GBM regions	[27,33,52]
	Farnesyl diphosphate	CSF	LC-MS/MS-SRM	↑ GBM pre- vs. post-treatment	[28]
Phospholipids	Phosphatidylserine (38:9)	Plasma	ESI-LTQ-MS	↑ Associated with IDH-wt GBM	[21]
	Phosphatidylcholine	Tumor	LC-MS/MS HR-MAS	↑ Associated with poor OS ↑ Associated with GBM	[34,36]
	Lyso PC a C18:0	Plasma	FIA-MS	↑ GBM patients vs. healthy individuals ↑ Associated with poor OS	[26]
	Lyso PC a C16:0 Lyso PC a C18:1 Lyso PC a C20:3 PC aa C38:5 PC ae C42:5	Plasma	FIA-MS	↑ Associated with poor OS	[26]
	PC aa C14:2	Tumor	LC-MS/MS	↓ GBM patients vs. healthy individuals	[27]
	PC ae C40:3	Plasma	FIA-MS	↑ GBM patients vs. healthy individuals	[26]
	PC ae C40:6	Tumor	LC-MS/MS	↑ GBM patients vs. healthy individuals	[27]
	PC aa C36:5	Plasma	FIA-MS	↓ GBM patients vs. healthy individuals	[26]
	PC aa C36:4	Plasma	FIA-MS	↑ Associated with better OS	[26]
	PC aa C38:6 PC aa C34:1	Plasma	LC-MS/MS	↑ GBM patients vs. healthy individuals	[27]
	PC aa C32:1	Plasma Tumor	FIA-MS MALDI-TOF	↓ GBM patients vs. healthy individuals ↑ GBM patients vs. healthy individuals	[26,27]
	O-phosphoethanolamine	Plasma	GC-MS	↓ Glioma patients vs. healthy individuals	[24]
Triglycerids	Triglycerol [48:1, 48:2, 50:2, 50:3, 52:2, 52:3, 52:4, 52:5, 54:3, 54:4, 54:5, 54:6]	Plasma	LC-MS/MS MALDI-TOF	↑ GBM patients vs. healthy individuals	[27]
Sphingolipids	3-O-sulfogalactosylceramide	Plasma	ESI-LTQ-MS	↓ Associated with IDH-wt GBM	[21]
	Sphingomyelin (33:1)	Tumor	MALDI-TOF	↑ GBM patients vs. healthy individuals	[27]

↑ denotes increased levels and ↓ denotes decreased levels of a specific metabolite. GBM, glioblastoma; LC-MS/MS, liquid chromatography–tandem mass spectrometry; MS, mass spectrometry; SRM, selected reaction monitoring; ESI, electrospray ionization; LTQ, linear trap quadrupole; HR-MAS, high-resolution magic-angle-spinning; MALDI, matrix-assisted laser desorption/ionization; FTICR, Fourier transform ion cyclotron resonance; TOF, time of flight; GC-MS gas chromatography–mass spectrometry; FIA, flow injection analysis, CSF, cerebrospinal fluid, IDH, isocitrate dehydrogenase; IDH-wt, isocitrate dehydrogenase wild-type; IDH-mut, isocitrate dehydrogenase mutant; OS, overall survival.

Glioblastoma cells rely on external uptake of cholesterol for their growth, proliferation, and invasion [27,33,52]. Glioblastoma cells also make a shift toward lipid oxidation pathways, utilizing FAs as an alternative energy source through β-oxidation [48]. This metabolic adaptation provides ATP and supports the biosynthesis of macromolecules necessary for tumor growth. Targeting enzymes involved in FA oxidation has shown potential in lowering glioblastoma cell viability [48,53]. We also cannot forget glioblastoma’s ability to quickly shift between lipid metabolism and other metabolic pathways if the need arises. For instance, mutations in IDH1 can influence lipid metabolism by affecting NADPH production, which is crucial for FA synthesis and redox balance [54].

### 2.4. Metabolism of Nucleotides, Vitamins, and Hormones

Recent metabolomic studies have also identified significant alterations in nucleotide, vitamin, hormone, and redox balance in glioblastoma, providing potential biomarkers for diagnosis and prognosis. In nucleotide metabolism, elevated levels of adenine and adenosine monophosphate (AMP) in tumor tissues have been linked to poor overall survival in glioblastoma patients (shown in Table 4) [34]. Conversely, decreased levels of deoxyguanosine monophosphate (dGMP) are also associated with unfavorable outcomes. Metabolites such as uracil, thymine, uridine, and deoxyinosine show increased concentrations in IDH wildtype (IDH-wt) glioblastoma, while guanosine levels are reduced in these cases [25,34]. A recent study by Zhou et al. found a link between purine metabolism and DNA repair therapy resistance in glioblastoma. By supplementing glioblastoma with external purines, they enhanced their radiation-resistant capability, while depleting purines led to a more radiation-susceptible glioblastoma model. Based on this finding, targeting purine metabolism pathways could improve treatment outcomes when targeting glioblastoma DNA repair mechanisms [55].

**Table 4:** Nucleotide, vitamin, and metabolic biomarker changes in glioblastoma. Overview of recent metabolomics studies performed on tumor tissue and plasma and CSF samples.

Molecules	Metabolites	Sample Type	Identification Method	GBM Specificity	Ref.
Nucleotide and nucleic acid metabolism	Adenine	Tumor	LC-MS/MS	↑ Associated with poor OS	[34]
	Uracil	Tumor Plasma	LC-MS/MS	↑ Associated with IDH-wt GBM ↑ Associated with high-grade gliomas	[25,34]
	Thymine	Tumor	LC-MS/MS	↑ Associated with IDH-wt GBM	[34]
	Uridine	Tumor CSF Plasma	LC-MS/MS MSI LC-MS/MS-SRM LC-MS/MS	↑ Associated with IDH-wt GBM ↑ GBM vs. peritumoral tissue ↑ GBM pre- vs. post-treatment	[3,25,28,34]
	Deoxyinosine	Tumor	LC-MS/MS	↑ Associated with IDH-wt GBM	[34]
	Guanosine	Tumor	LC-MS/MS	↓ Associated with IDH-wt GBM	[34]
	AMP	Tumor	LC-MS/MS MSI	↑ Associated with poor OS ↑ GBM vs. peritumoral tissue	[3,34]
	ADP UMP UDP	Tumor	MSI	↑ GBM vs. peritumoral tissue	[3]
	dGMP	Tumor	LC-MS/MS	↓ Associated with poor OS	[34]
	dCMP Nicotinamide mononucleotide	Tumor	LC-MS/MS	↓ Associated with IDH-wt GBM	[34]
	5-hydroxymethyluracil	Plasma	ESI-LTQ-MS	↑ Associated with IDH-wt GBM	[21]
Vitamins, hormones, redox metabolism	Ascorbic acid Glutathione	Tumor	MALDI-FTICR-MS	↑ GBM core vs. peritumoral tissue	[3]
	Thiamine	Tumor	LC-MS/MS	↑ Associated with IDH-wt GBM	[34]
	Pyridoxal phosphate	Tumor	LC-MS/MS	↓ Associated with IDH-wt GBM	[34]
	N-acylphosphatidylethanolamine	Plasma	ESI-LTQ-MS	↑ Associated with IDH-wt GBM	[21]
	Choline	CSF Plasma Tumor	LC-MS/MS-SRM H-NMR HR-MAS	↑ TP53-wt vs. TP53-mut GBM ↑ PTEN-mut vs. PTEN-wt GBM ↓ Glioma patients vs. healthy individuals ↑ Associated with GBM	[23,28,36]
Other	Shikimate	CSF	LC-MS/MS-SRM	↑ GBM pre- vs. post-treatment	[28]
	Trimethylamine-N-oxide	Plasma	LC-MS/MS	↑ Associated with IDH-wt gliomas	[25]

↑ denotes increased levels and ↓ denotes decreased levels of a specific metabolite. GBM, glioblastoma; LC-MS/MS, liquid chromatography–tandem mass spectrometry; MS, mass spectrometry; SRM, selected reaction monitoring; ESI, electrospray ionization; LTQ, linear trap quadrupole; HR-MAS, high-resolution magic-angle-spinning; H-NMR, proton nuclear magnetic resonance; MALDI, matrix-assisted laser desorption/ionization; FTICR, Fourier transform ion cyclotron resonance; MSI, mass spectrometry imaging; CSF, cerebrospinal fluid, IDH, isocitrate dehydrogenase, IDH-wt, isocitrate dehydrogenase wild-type; OS, overall survival.

Ascorbic acid and glutathione are elevated in glioblastoma core tissues relative to peritumoral regions, suggesting enhanced oxidative stress responses. Thiamine levels rise in IDH-wt glioblastoma, whereas pyridoxal phosphate decreases [3,34]. In addition to their use as potential biomarkers, vitamins have also been proposed as potential adjuvant treatments for combating glioblastoma. Throughout the literature, vitamin D stands out, as it has shown an anti-tumor protective effect by inducing cell cycle arrest and cell death in glioblastoma [56]. Retinoids, vitamin E, and ascorbic acid were also observed to have an anti-cancer effect in glioblastoma, though this area of research is still highly contested [57,58].

Researchers also found some other interesting metabolites that were differentially expressed in glioblastoma, in particular, shikimate in CSF. Shikimate is an intermediate of the shikimate pathway, which is not naturally present in animals but can be found, among other organisms, in microorganisms living in the human gut microbiome. This finding suggests a possible relationship with the gut microbiome, in particular *Akkermansia* sp., which elevates the shikimate intermediate in the CSF of patients with glioblastoma [28]. To propose a more solid idea of the connections between glioblastoma and the gut microbiome, more research needs to be done.

## 3. Metabolomics Approaches in Glioblastoma

### 3.1. Targeted and Untargeted Approaches

**Figure 2:**
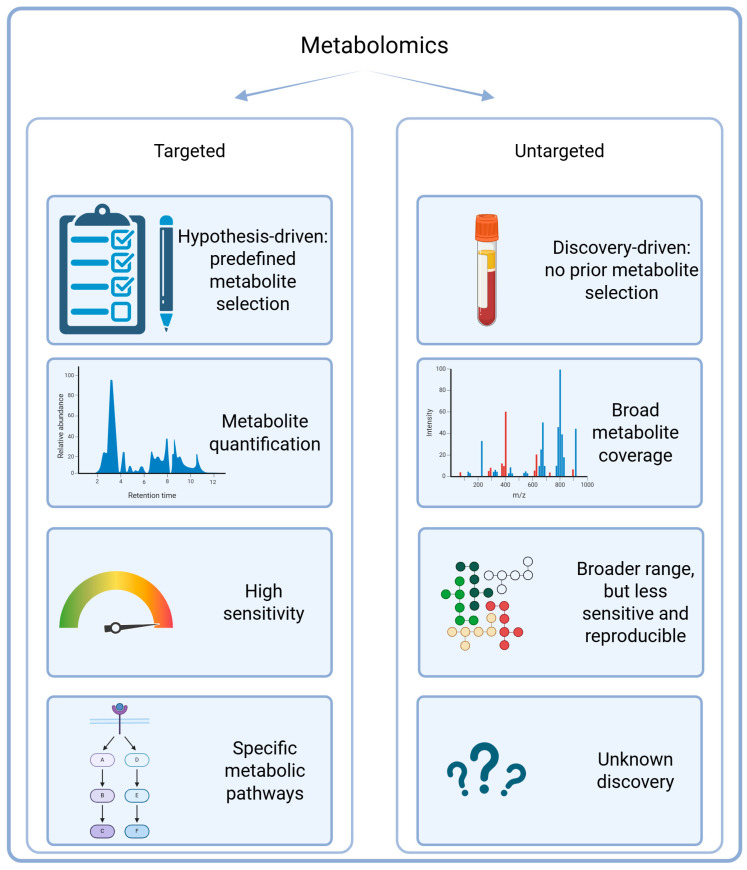
Comparison of targeted and untargeted metabolomics approaches highlighting key features. Created in BioRender. Videtič Paska, A. (2025) https://BioRender.com/7vhpmxv.

Targeted and untargeted metabolomics follow distinct experimental strategies (Figure 2). Targeted strategies focus on the precise quantification of selected numbers of metabolites, often from the same metabolic pathways. Targeted methods are usually more straightforward, with lower limits of detection and easier analysis and interpretation [59]. Untargeted metabolomics methods are a more discovery-driven approach that enable comprehensive profiling without prior selection of specific metabolites. Untargeted methods capture broad metabolic changes, enabling the discovery of multiple novel biomarkers and changed metabolic pathways associated with disease states like glioblastoma [60,61].

#### 3.1.1. Overview of Targeted Approaches

**Figure 3:**
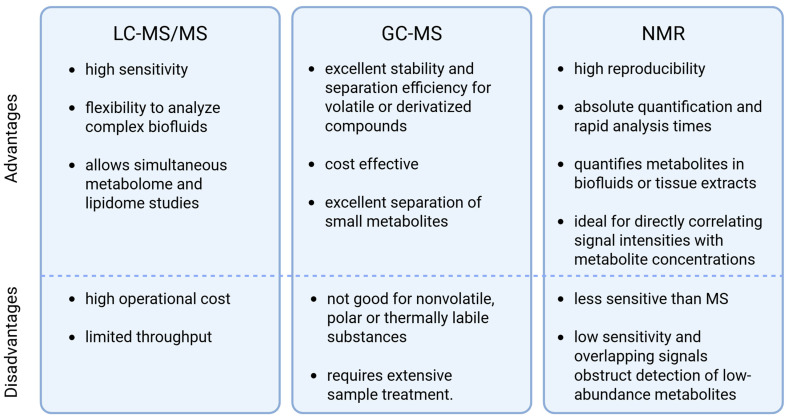
Advantages and disadvantages of the most commonly used targeted analytical platforms. Image adapted according to [64,65]. LC-MS/MS, liquid chromatography–tandem mass spectrometry; GC-MS, gas chromatography–mass spectrometry; NMR, nuclear magnetic resonance. Created in BioRender. Videtič Paska, A. (2025) https://BioRender.com/7lhp09e.

Targeted metabolomics is a hypothesis-driven approach that focuses on the measurement of predefined groups of known metabolites. Often, these metabolites are from metabolic pathways with relatively similar characteristics. Metabolites of interest are chosen, and optimized methods are developed for their extraction and detection. Well-defined targeted metabolomics methods typically include optimized isolation steps with experimentally determined efficiency and recovery from different biological matrices. Linearity, accuracy, dynamic range, limit of detection (LOD), and limit of quantification (LOQ) are determined for individual metabolites. In this way, the measurements can correlate as much as possible with the concentration of the analyte in the biological sample [60,62]. Common analytical platforms include LC-MS/MS, GC-MS, and NMR spectroscopy. For targeted LC-MS/MS assays, triple quadrupole instruments operating in multiple reaction monitoring (MRM) mode, which offers high sensitivity and selectivity for the chosen metabolites, are most commonly used [59,62,63]. GC-MS is employed for volatile or derivatized compounds, providing excellent separation for small metabolites (organic acids and amino acids). NMR can be applied in a targeted manner. One-dimensional ^1^H NMR or high-resolution magic-angle-spinning (HR-MAS) NMR can quantify specific metabolites in biofluids or tissue extracts. Their advantage is high reproducibility, but with lower sensitivity than MS methods [64]. Each method has its place, with advancements in methods aiming to overcome these limitations and enhance overall analytical performance, as presented in Figure 3 [65].

For targeted methods, calibration curves are prepared for each analyte, enabling absolute quantification of metabolite concentrations. By prioritizing known metabolites, targeted approaches maximize sensitivity, reproducibility, and quantification accuracy. Targeted approaches can provide clinically relevant results within hours, while untargeted analyses often require multiple days for data processing and metabolite identification. The use of defined extraction protocols and instrument settings minimizes variability, with reference standards reducing false identification. In sum, targeted metabolomics provides higher analytical precision and confidence in metabolite identification compared with untargeted approaches, at the cost of a lower number of metabolites [62,63].

#### 3.1.2. Overview of Untargeted Approaches

Untargeted metabolomics (also known as global or discovery metabolomics) aims to detect as many metabolites as possible in a sample without predetermined targets [61,62]. The goal is to capture a broad range of metabolites, including known and unknown metabolites. With this approach, the possibility to discover unexpected changes or novel biomarkers is higher. High-resolution mass spectrometry is the main method used for untargeted approaches. With accurate mass measurements and MS/MS fragmentation, the data enable the identification of metabolites post hoc [61]. Typically, samples are analyzed by LC-MS on instruments like time of flight (TOF) or Orbitrap MS, coupled with ultra-high-performance liquid chromatography (UPLC) for the best possible separation. These instruments can detect thousands of spectral features that represent an unbiased view of multiple metabolic pathways simultaneously [62,64]. Untargeted analyses benefit from complementary techniques to maximize coverage to confirm the findings using targeted methods, for example, using both positive and negative ionization modes, combining LC-MS with GC-MS, or adding NMR profiling for highly abundant metabolites [62]. New technologies like ion mobility spectrometry (IMS) are increasingly integrated with MS to enhance untargeted metabolomics. IMS separates ions based on their shape and charge in the gas phase. This adds an extra dimension of separation that helps resolve isomeric metabolites and reduce spectral complexity [60,66]. Overall, untargeted metabolomic methods are discovery-driven, and they favor broadness over absolute quantitation, typically reporting relative changes in metabolite abundances. The strength of untargeted metabolomics lies in its ability to capture broad metabolic changes without preconceived targets. By surveying hundreds to thousands of metabolites across diverse pathways, researchers can identify patterns and pathways that would be missed by a focused approach. The disadvantage of untargeted metabolomics is its complexity. The broad range of unknown metabolites often results in unclear identification. Additionally, there are challenges of lower reproducibility and validation. This can lead to false positive/negative detections and lower standardization of methods. The untargeted metabolomics approach is invaluable for hypothesis generation, since it can reveal unanticipated metabolic changes associated with disease or treatment [62].

#### 3.1.3. Data Processing and Analysis

In targeted metabolomics, data analysis is more straightforward, given that the focus is on predefined known metabolites. After data acquisition with LC-MS/MS, GC-MS, or NMR, preprocessing involves peak detection, integration, and normalization using internal standards for variability correction [63]. The obtained signal is transformed to the concentration of metabolites using calibration curves of standard solutions. Data are then subjected to conventional statistical tests (e.g., *t*-tests, ANOVA) to determine significant differences in metabolite levels [59]. This focused workflow minimizes complexity and yields highly confident, reproducible results that are critical for validating biomarkers in diseases such as glioblastoma.

Untargeted metabolomics generates large, complex datasets that require more preprocessing, applying advanced statistical methods for analysis, usually by highly trained experts in the field. Data from high-resolution LC-MS/MS, GC-MS, or NMR undergoes steps such as peak detection, deconvolution, retention time alignment, and normalization to account for instrumental drift. Feature extraction is followed by metabolite identification using spectral libraries and databases like HMDB [67] and METLIN [68]. Subsequent statistical analysis often employs multivariate methods (e.g., PCA, PLS-DA) and machine learning algorithms to uncover patterns and distinguish specific metabolic profiles. Pathway enrichment analysis helps place these findings in a broader biological context [61].

### 3.2. Multiomics Integration

“Omic” technologies, including genomics, transcriptomics, proteomics, and metabolomics, are essential approaches in the search for new biomarkers in all complex diseases. Metabolomics represents the downstream step of all these other omics, and therefore, provides a functional readout of upstream omic alterations and a more direct explanation of (changes in the) processes in the organism. Through a multiomics perspective, more holistic interrogation of potential biomarkers is possible, as they enable the observation of cellular processes from different perspectives, and at the same time, can diminish the biological and experimental bias effects. However, due to the massive amount of data, the major drawback lies in the interpretation of the results. Data analysis and its integration is based on the use of network and clustering-based models [69].

To our knowledge, there is a rather limited number of studies in which metabolomics and any of the previously mentioned omic approaches were applied and the results evaluated at the same time. Among the most recent studies using the multiomic approach is the GLIOPLAK trial [26]. Its metabolome results are presented in more detail in Table 2 and Table 3. The study targeted plasma metabolome and proteome analyses from unresected glioblastoma patients that were treated with radiotherapy and temozolomide, and it showed that patients’ circulating omic profiles differed from those of healthy subjects. Out of 265 differentially expressed proteins and metabolites, 5 metabolites (pimeloylcarnitine, leucine, asparagine, lysoPC a C18:0, PC ae C40:3) and 5 proteins (NPY, KLK13, SCLY, S100A4, CXCL17) showed AUCs above 0.74. The combination of these molecules most of the times correctly classified the patients and controls to their corresponding groups. Additionally, some of the omic attributes showed potential for use as prognostic or diagnostic markers [26].

Migliozzi et al. [70] used a glioblastoma dataset from the Clinical Proteomic Tumor Analysis Consortium and reconstructed functional subtypes of glioblastoma using proteomics, phospho-proteomics, acetylomics, metabolomics, and lipidomics data. The four subtypes of IDH-wt glioblastoma were classified into two functional branches: neurodevelopment (proliferative/progenitor (PPR) and neuronal (NEU)) and metabolism (glycolytic/plurimetabolic (GPM) and mitochondrial (MTC)). For the both the GPM and MTC subtypes, higher levels of glycolytic enzymes and lower levels of mitochondrial enzymes (translocases, TCA cycle, and electron transport chain enzymes) were found. The post-translational modification lysine acetylation was highest on mitochondrial enzymes in the GPM subtype, while hyperacetylation in the MTC subtype was associated with enzymes linked to glycolysis and the pentose phosphate pathway, amino acid biosynthesis, and adipogenesis. Part of the PPR subtype samples showed the highest acetylation in histone and non-histone acetyltransferases and in other chromatin remodeling, DNA damage response, and DNA replication stress enzymes. These PPR samples had the highest proliferation and stemness scores. When analyzing metabolites, the GPM subtype showed higher abundance with intermediates of glycolysis, the pentose phosphate shunt, fatty acids, sugars, and essential amino acids. The MTC subtype showed elevated TCA cycle intermediates, antioxidants, and non-essential amino acids. The lipidomic data showed abundance of triacylglycerol in the GPM subtype, while MTC glioblastoma was associated with increased acyl-carnitine and diacylglycerol. The PPR subtype had enriched phosphatidylcholines, and the NEU subtype accumulated sphingomyelin, phosphatidylserine, hexosyl-ceramide and cholesteryl ester, and phosphatidic acid [70].

A novel approach that enables spatial correlation lipidomic and proteomic LC-MS/MS analysis was established by Hendriks et al. [71]. Due to the heterogeneous nature of the glioblastoma tissues, the spatially linked information allows thorough understanding of the molecular environment and the interactions of the lipid–protein network. The comparison of the tumor and necrotic regions by interaction network analysis was performed on the significantly changed protein-coding genes and lipids. Differences in up- and downregulated proteins and lipids were determined and were linked to glycerolipid and glycerophospholipid metabolism; sphingolipid metabolism; arginine biosynthesis; and nicotinate, nicotinamide, and pyruvate metabolism [71].

Barzegar Behrooz et al. [72] performed an extensive multiomic analysis on information available from different databases on gene expression, miRNA expression, and from recent studies on metabolites. They identified 11 genes (*UBC*, *HDAC1*, *CTNNB1*, *TRIM28*, *CSNK2A1*, *RBBP4*, *TP53*, *APP*, *DAB1*, *PINK1*, and *RELN*), 5 miRNAs (hsa-mir-221-3p, hsa-mir-30a-5p, hsa-mir-15a-5p, hsa-mir-130a-3p, and hsa-let-7b-5p), 6 metabolites (HDL, N6-acetyl-L-lysine, cholesterol, formate, N, N-dimethylglycine/xylose, and X2. piperidinone), and 15 specific signaling pathways that could be designated as fundamental in glioblastoma development and could be further used for the design of diagnostic approaches and personalized treatments.

Differences in metabolic changes in the brain are important also in the light of the application of different drugs. Multiomic analysis of treatment with paclitaxel and/or topotecan was performed on the cell line U87. This showed differences in protein and metabolite expression linked to particular molecular mechanisms associated with the chemotherapy response [73].

## 4. Clinical Translation of Metabolomics

Multimodal analyses are slowly becoming standard in cancer research and detection. However, because of limitations regarding sample size and amount, combining various techniques is not always feasible. Therefore, the use of metabolomics that use a range of samples from liquid to classical biopsy may provide novel opportunities for cancer research, detection, and monitoring. Metabolites reflect metabolic processes, taking into consideration genetic and environmental factors [65]. Because of this, the implementation of metabolomics shows promise in the field of personalized medicine, as it can provide information about patients’ responses. Compared with genomics, which provides patterns that can be assigned to specific diseases, metabolomics provides information about modifications in metabolites, which is “closer” to the disease state. An upgrade in personalized medicine could be the integration of pharmacometabolomics, which takes into consideration the pharmacokinetic and pharmacodynamic drug processes together with metabolite profiling [74]. This branch of metabolomics can help in the prediction of drug response based on the interaction between the pharmacology of the drug and the pathophysiology of the patient. Metabolomics is more diagnostic, determining changes in tumor biochemistry, while pharmacometabolomics can be considered a prognostic methodology.

However, when it comes to clinical applications, the interpretation and comparison of data is a limitation. Moreover, method standardization has not reached a level suitable for use in clinical settings. Finally, reference datasets for metabolites must be generated so the biomarker discovery will not depend on comparisons of patients with selected healthy controls. This can be challenging, both because of the different environmental influences and because of age and gender differences among the analyzed groups. For implementation of metabolomics in clinical settings, it may be beneficial to opt for interdisciplinary teams consisting of physicians, researchers, and data analysts that can provide proper sample handling (preparation and processing) but also timely results for precise diagnosis.

## 5. Strengths and Limitations

Metabolomics offers us high sensitivity and specificity in detecting and quantifying metabolites, especially when targeted. It also provides insights into cellular processes and disease mechanisms that were not visible before [3,75]. Metabolomics is particularly valuable in oncology, neurology, and metabolic disorders, where subtle metabolic shifts can indicate disease onset, progression, or response to treatment [75]. By analyzing the unique metabolic markers of glioblastoma, researchers can identify biomarkers for early detection, prognosis, and monitoring of treatment responses. Table 1, Table 2, Table 3 and Table 4 give an overview of elevated as well as reduced levels of certain tissue, plasma, and CSF metabolites that have been differentially expressed in glioblastoma patients in recent studies. The findings underscore the potential of metabolomics in clinical applications [6,21,23,24]. Metabolic profiling of glioblastoma also highlights dysregulated metabolite pathways, which can be exploited in personalized treatments. The studies summarized in Table 1, Table 2, Table 3 and Table 4 have identified changes in amino acid metabolism, lipid profiles, and energy production pathways, providing a deeper understanding of tumor metabolism [21,76]. The integration of metabolomics with other omics technologies enhances the precision of biomarker discovery and enables the identification of novel therapeutic targets. The changes between metabolite levels and genomic alterations, for instance IDH mutations and TP53 mutations, also provide a framework for personalized medicine [28,42].

There are numerous research studies that are currently investigating metabolites which are dysregulated in glioblastomas. Some of the more interesting ones are of lactate [6,24,28], carnitine [25,27,34,42], myo-inositol [23,31,36,38,39], and 2-hydroxyglutarate [3,13,29,32,34,35,36], which could serve as promising biomarkers for monitoring tumor status and therapeutic response, but are not glioblastoma-specific. Currently, 2-hydroxyglutarate serves as an oncometabolite found in cancers like kidney, hematopoietic, and neurocrine cancer, in which its mutation promotes HIF-1 and mTOR signaling pathway alterations as well as DNA repair disruptions [37]. Since an overexpression of 2-hydroxyglutarate is a direct result of the IDH gene mutation, 2-hydroxyglutarate currently stands as an excellent metabolic predictor of IDH-mutated gliomas or low-grade gliomas [77]. Carnitine, on the other hand, is an amino acid derivative that protects cells against oxidative stress and stabilizes membranes. Many treatment-resistant glioblastoma tissue samples were found containing upregulated OCTN2 gene, which metabolizes and activates carnitine. The link was confirmed by Fink et al., associating carnitine and poor glioblastoma patient survival [78]. Carnitine dysregulation is also associated with cancers like acute myeloid leukemia and ovarian, breast, lung, endometrial, renal, pancreatic, and colon cancer [79]. Multiple cancers are also associated with higher lactate and lower myo-inositol levels, which help them grow and proliferate [80,81]. Furthermore, elevated levels of specific amino acids like taurine, citrulline, and serine predicted poor OS in one study, which could offer prognostic insights [26]. Another study found glycerol-3-phosphate and phosphatidylcholine as potential target metabolites that could be exploited when targeting glioblastoma metabolism [82]. Studies also pointed out how metabolomics and metabolites like N-acetylputrescine, carnitine, farnesyl diphosphate, and uridine could be utilized in monitoring treatment responses by identifying metabolic shifts that occur as a result of therapy. This approach enables the evaluation of therapeutic efficacy and allows for the early detection of resistance mechanisms, guiding treatment decisions and enhancing patient outcomes [28,42].

Metabolomics research is not without its limitations, however. Inconsistencies in the data are evident, as demonstrated by discrepancies in lactate levels between studies. Kelimu et al. observed decreased plasma lactate levels, whereas Löding et al. reported increased lactate levels in glioma patients compared with healthy individuals [23,24]. Such variations in metabolite levels, even when analyzing the same sample type, can result from differences in experimental design, patient heterogeneity, and technical methodologies, adding complexity to the interpretation of findings. These inconsistencies highlight the critical need for standardized protocols and larger, more diverse patient cohorts to improve the reproducibility and accuracy of metabolomic studies in glioblastoma [75].

Despite these challenges, metabolomics remains a promising approach in advancing our understanding of glioblastoma, offering a robust platform for biomarker discovery and the identification of novel therapeutic targets. Its application in clinical settings has the potential to enhance early detection, personalize treatment strategies, and improve prognostic evaluations, ultimately contributing to better patient care.

## 6. Conclusions

For decades, the identification of metabolic vulnerabilities in cancer that can be used in targeted therapeutic approaches has been a topic of interest. Even though most research has been focused on glucose metabolism, the ability of brain tumor cells to use other substrates such as KBs or FAs to ensure their growth has become apparent. Targeting metabolites to attack glioblastoma cells has not yet yielded results. For this to happen, it is crucial to deepen our understanding of the changes in metabolic processes that lead to glioblastoma occurrence, progression, and invasion. Defining metabolic signatures can be used for risk stratification, outcome prediction, and in the design of metabolic-targeting therapies.

Analyzing metabolites from blood or plasma should be considered as diagnostic and prognostic tools especially because of their non-invasive character. It is also important to consider the metabolite composition of CSF, which can provide clinically relevant biomarkers or even a new basis of understanding the pathophysiology of glioblastoma.

## Data Availability

No new data were created or analyzed in this study.
